# A comparison of four serological assays for detecting anti–SARS-CoV-2 antibodies in human serum samples from different populations

**DOI:** 10.1126/scitranslmed.abc3103

**Published:** 2020-09-02

**Authors:** Ludivine Grzelak, Sarah Temmam, Cyril Planchais, Caroline Demeret, Laura Tondeur, Christèle Huon, Florence Guivel-Benhassine, Isabelle Staropoli, Maxime Chazal, Jeremy Dufloo, Delphine Planas, Julian Buchrieser, Maaran Michael Rajah, Remy Robinot, Françoise Porrot, Mélanie Albert, Kuang-Yu Chen, Bernadette Crescenzo-Chaigne, Flora Donati, François Anna, Philippe Souque, Marion Gransagne, Jacques Bellalou, Mireille Nowakowski, Marija Backovic, Lila Bouadma, Lucie Le Fevre, Quentin Le Hingrat, Diane Descamps, Annabelle Pourbaix, Cédric Laouénan, Jade Ghosn, Yazdan Yazdanpanah, Camille Besombes, Nathalie Jolly, Sandrine Pellerin-Fernandes, Olivia Cheny, Marie-Noëlle Ungeheuer, Guillaume Mellon, Pascal Morel, Simon Rolland, Felix A. Rey, Sylvie Behillil, Vincent Enouf, Audrey Lemaitre, Marie-Aude Créach, Stephane Petres, Nicolas Escriou, Pierre Charneau, Arnaud Fontanet, Bruno Hoen, Timothée Bruel, Marc Eloit, Hugo Mouquet, Olivier Schwartz, Sylvie van der Werf

**Affiliations:** 1 Virus and Immunity Unit, Department of Virology, Institut Pasteur, CNRS UMR 3569, Paris, France.; 2 Vaccine Research Institute, Creteil, France.; 3 Université de Paris, Sorbonne Paris Cité, Paris, France.; 4 Pathogen Discovery Laboratory, Department of Virology, Institut Pasteur, Paris, France.; 5 Laboratory of Humoral Immunology, Department of Immunology, Institut Pasteur, INSERM U1222, Paris, France.; 6 Molecular Genetics of RNA Viruses, Department of Virology, Institut Pasteur, CNRS UMR 3569, Paris, France.; 7 Université de Paris, Paris, France.; 8 Emerging Diseases Epidemiology Unit, Department of Global Health, Institut Pasteur, Paris, France.; 9 Department of Virology, Institut Pasteur, Paris, France.; 10 National Reference Center for Respiratory Viruses, Institut Pasteur, Paris, France.; 11 RNA Biology of Influenza Virus, Department of Virology, Institut Pasteur, Paris, France.; 12 Pasteur-TheraVectys joined unit, Institut Pasteur, Paris, France.; 13 Molecular Virology and Vaccinology Unit, Department of Virology, Institut Pasteur, Paris, France.; 14 Innovation lab: Vaccines, Institut Pasteur, Paris, France.; 15 Plate-Forme Technologique Production et Purification de Protéines Recombinantes, Institut Pasteur, Paris, France.; 16 Structural Virology Unit, Department of Virology, Institut Pasteur, CNRS UMR 3569, Paris, France.; 17 Université of Paris, INSERM UMR 1137 IAME, Paris, France.; 18 Medical and Infectious Diseases Intensive Care Unit, Assistance Publique-Hôpitaux de Paris, Bichat–Claude-Bernard University Hospital, Paris, France.; 19 Department of Virology, Assistance Publique-Hôpitaux de Paris, Bichat–Claude-Bernard University Hospital, Paris, France.; 20 Department of Infectious Diseases, Assistance Publique-Hôpitaux de Paris, Bichat–Claude-Bernard University Hospital, Paris, France.; 21 Department of Epidemiology, Biostatistics and Clinical Research, Assistance Publique-Hôpitaux de Paris, Bichat–Claude-Bernard University Hospital, INSERM CIC-EC 1425, Paris, France.; 22 Investigation Clinique et Accès aux Ressources Biologiques (ICAReB), Center for Translational Research, Institut Pasteur, Paris, France.; 23 Unité Coordination du Risque Epidémique et Biologique, AP-HP, Hôpital Necker, Paris, France.; 24 Etablissement Français du Sang (EFS), Paris, France.; 25 Service de maladies infectieuses, hôpital universitaire Cavale Blanche, Brest, France.; 26 CIC 1417, CIC de vaccinologie Cochin-Pasteur, AP-HP, Hôpital Cochin, Paris, France.; 27 Direction alerte et crises, réserve sanitaire, Santé publique France, Saint-Maurice, France.; 28 Centre d’épidémiologie et de santé publique des armées, Marseille, France.; 29 Direction Générale de la Santé, Paris, France.; 30 PACRI Unit, Conservatoire National des Arts et Métiers, Paris, France.; 31 Direction de la recherche médicale, Institut Pasteur, Paris, France.; 32 National Veterinary School of Alfort, Maisons-Alfort, France.

## Abstract

It is of paramount importance to evaluate the prevalence of both asymptomatic and symptomatic cases of SARS-CoV-2 infection and their differing antibody response profiles. Here, we performed a pilot study of four serological assays to assess the amounts of anti–SARS-CoV-2 antibodies in serum samples obtained from 491 healthy individuals before the SARS-CoV-2 pandemic, 51 individuals hospitalized with COVID-19, 209 suspected cases of COVID-19 with mild symptoms, and 200 healthy blood donors. We used two ELISA assays that recognized the full-length nucleoprotein (N) or trimeric spike (S) protein ectodomain of SARS-CoV-2. In addition, we developed the S-Flow assay that recognized the S protein expressed at the cell surface using flow cytometry, and the luciferase immunoprecipitation system (LIPS) assay that recognized diverse SARS-CoV-2 antigens including the S1 domain and the carboxyl-terminal domain of N by immunoprecipitation. We obtained similar results with the four serological assays. Differences in sensitivity were attributed to the technique and the antigen used. High anti–SARS-CoV-2 antibody titers were associated with neutralization activity, which was assessed using infectious SARS-CoV-2 or lentiviral-S pseudotype virus. In hospitalized patients with COVID-19, seroconversion and virus neutralization occurred between 5 and 14 days after symptom onset, confirming previous studies. Seropositivity was detected in 32% of mildly symptomatic individuals within 15 days of symptom onset and in 3% of healthy blood donors. The four antibody assays that we used enabled a broad evaluation of SARS-CoV-2 seroprevalence and antibody profiling in different subpopulations within one region.

## Elucidating seroprevalence in COVID-19

Individuals infected with SARS-CoV-2 mount an antibody response that may vary depending on the severity of the disease. Grzelak *et al.* assessed the amounts of anti–SARS-CoV-2 antibodies in serum samples from 491 healthy individuals before the pandemic, 51 individuals hospitalized with COVID-19, 209 suspected cases of COVID-19 with mild symptoms, and 200 healthy blood donors. The authors developed and compared the performance of four different serological assays measuring antibody amounts and their neutralizing activity. The assays enabled a broad evaluation of SARS-CoV-2 seroprevalence and antibody profiling in asymptomatic, mildly symptomatic, and severe cases of COVID-19.

## Teaser

Different serological assays measuring anti–SARS-CoV-2 antibodies and their neutralizing activity in samples from individuals with severe and mild COVID-19 are compared.

## INTRODUCTION

Within a few months of the initial description of atypical pneumonia cases in Wuhan in December 2019, coronavirus disease 2019 (COVID-19) became a major pandemic threat. As of 8 August 2020, about 20 million infections have been officially diagnosed, with 0.7 million fatalities worldwide. COVID-19 is caused by severe acute respiratory syndrome coronavirus 2 (SARS-CoV-2) (*1*) (*2*), a betacoronavirus displaying 80% nucleotide homology with severe acute respiratory syndrome virus (SARS-CoV), which was responsible for an outbreak of 8000 estimated cases of SARS in 2003.

Polymerase chain reaction (PCR)–based tests are widely used to diagnose COVID-19 and for detection and quantification of SARS-CoV-2 RNA (*3*) (*4*) (*5*). These tests are crucial for monitoring individuals with active SARS-CoV-2 infection. The average virus RNA load is 10^5^ copies per nasal or oropharyngeal swab sample 5 days after symptom onsets and may reach 10^8^ copies (*6*). A decline in SARS-CoV-2 RNA occurs 10 to 11 days after symptom onset, but viral RNA can be detected for more than 28 days after symptom onset in recovered patients at a time when anti–SARS-CoV-2 antibodies are readily detectable (*6*)(*7*). Disease severity correlates with viral load, and elderly patients, who are particularly susceptible to infection, generally display higher viral loads than do younger individuals (*6*)(*7*).

A number of different serological assays have been developed. These assays usually detect anti-spike (S) protein or anti-nucleoprotein (N) antibody responses in those with COVID-19, because the two proteins are highly immunogenic. The viral S protein enables the virus to enter target host cells by binding to a cellular receptor, angiotensin-converting enzyme 2 (ACE2) for SARS-CoV-2 (and also SARS-CoV). Virus entry is followed by cleavage of S and priming by the cellular protease TMPRSS2 or other endosomal proteases (*8*). The S proteins from SARS-CoV and SARS-CoV-2 share 76% amino acid similarity (*2*). One notable difference between the two viruses is the presence of a furin cleavage site in SARS-CoV-2 S protein, which is suspected to enhance viral infectivity (*2*). The structures of the S protein from SARS-CoV and SARS-CoV-2 in complex with ACE2 have been elucidated (*9*–*11*). The S protein consists of three S1-S2 dimers displaying different conformational changes upon virus entry, leading to fusion of the virus with host cell membranes (*9*, *10*, *12*). Some anti-S antibodies, including those targeting the receptor binding domain (RBD) of the S protein, display neutralizing activity, but their relative frequency among anti–SARS-CoV-2 antibodies generated during infection remains poorly characterized. Nucleoprotein (N) is highly conserved between SARS-CoV-2 and SARS-CoV (96% amino acid homology). N plays a crucial role in subgenomic viral RNA transcription and viral replication and assembly.

Serological assays are usually performed using in-house or commercially available enzyme-linked immunosorbent assay (ELISA)–based diagnostics tests (*6*, *7*, *13*–*15*). Other techniques, including point-of-care tests, are also available. In hospitalized individuals with COVID-19, seroconversion is typically detected between 5 and 14 days after symptom onset, with a median time of 5 to 12 days for anti-S immunoglobulin M (IgM) antibodies and 14 days for anti-S IgG and IgA antibodies (*6*, *7*, *13*–*16*). The kinetics of anti-N antibody responses are similar to those of anti-S antibody responses, although N responses might appear earlier (*15*–*17*). Anti–SARS-CoV-2 antibody titers correlate with disease severity, likely reflecting higher viral replication rates and immune activation in patients with severe disease. Besides anti-N and anti-S antibodies, antibody responses to other viral proteins (ORF9b and NSP5) have also been identified by antibody microarray assays (*17*).

Antibody neutralization titers observed in individuals infected with other coronaviruses, such as Middle East respiratory syndrome coronavirus (MERS-CoV), are considered to be relatively low (*6*, *18*). With SARS-CoV-2 infection, neutralizing antibodies have been detected in symptomatic individuals (*6*, *8*, *19*, *20*), and their potency seems to be associated with high amounts of these antibodies. Neutralization is assessed using plaque neutralization assays, microneutralization assays, or inhibition of infection assays using pseudotype virus carrying the SARS-CoV-2 S protein (*6*, *8*, *19*–*21*). Potent neutralizing monoclonal antibodies that target the RBD of the S protein have been cloned from individuals infected with SARS-CoV-2 (*22*). Whether asymptomatic SARS-CoV-2 infections, which are not well characterized (*23*), can lead to protective immunity and whether this immunity is mediated by neutralizing antibodies remain crucial questions.

Here, we have designed anti-N antibody and anti-S antibody ELISA assays as well as two new assays for detecting anti–SARS-CoV-2 antibodies and their virus neutralization capabilities. We compared their performance and carried out anti–SARS-CoV-2 antibody profiling of different population subsets from the same region.

## RESULTS

### Measuring anti–SARS-CoV-2 antibodies in human serum samples with two different ELISA assays

We first designed four different serological assays to measure anti–SARS-CoV-2 antibodies in human serum samples from individuals with COVID-19 who were hospitalized or mildly symptomatic (Table 1). Two of the assays were standard ELISA assays using as target antigens the full-length nucleocapsid (N) protein (ELISA N) or the extracellular domain of the S protein in the form of a trimer (ELISA tri-S). The two recombinant antigens were produced in *Escherichia coli* (N) or in human cells (S) in vitro.

**Table 1:** The four serological assays used in this study.

**Assay**	**Antigen**	**Serum dilution**	**Readout**
ELISA N	N	1:200	Optical density
ELISA tri-S	Trimeric S	1:400	Optical density
S-Flow	S at the cell surface	1:300	Flow cytometry
LIPS	S1 and N	1:10	Bioluminescence (luciferase)

The ELISA N assay is an indirect test for the detection of total Ig, using 96-microwell plates coated with a purified His-tagged SARS-CoV-2 N protein. Titration curves of serum samples from 22 individuals with COVID-19 and four pre-pandemic human serum samples led to the determination that a dilution of 1:200 was of optimal sensitivity and specificity, and this dilution was then used for testing serum samples from larger cohorts. The ELISA tri-S assay enabled the detection of IgG antibodies directed against the SARS-CoV-2 S protein ectodomain. The ELISA tri-S assay has as antigen a purified, recombinant, and tagged form of the S protein ectodomain, which was stabilized and trimerized using a foldon motif. Serum IgG antibodies in serum samples from 100 healthy individuals before pandemic, 209 mildly symptomatic individuals suspected of having COVID-19, and 51 hospitalized patients with COVID-19 were titrated using serum dilutions ranging from 1:100 to 1:1,638,400 (fig. S1). Receiver operating characteristic (ROC) curves using either the total area under the curve (AUC) or single optical density (OD) measurements indicated that the 1:400 dilution provided the best sensitivity and specificity values and was therefore used in subsequent analyses (fig. S1). The ELISA tri-S assay also permitted the titration of anti-S IgM and anti-S IgA antibodies in human serum samples (fig. S1).

### Measuring anti–SARS-CoV-2 antibodies in human serum samples with the S-Flow assay

The third assay that we used, termed the S-Flow assay, is based on the recognition of SARS-CoV-2 S protein expressed on the surface of 293T cells (293T-S). We reasoned that in situ expression of the SARS-CoV-2 S protein would allow detection of antibodies binding to various conformations and domains of the viral S protein (fig. S2A). We verified that the S protein expressed on the surface of 293T cells was functionally active by mixing 293T-S cells with target cells expressing the ACE2 receptor. Large syncytia were detected, indicating that the S protein bound to its ACE2 receptor resulting in viral and host cell membrane fusion. 293T-S cells were incubated with dilutions of human serum samples, and antibody binding was detected by adding a fluorescent secondary antibody (anti-IgG or anti-IgM antibody). The signal was measured by flow cytometry using an automated 96-well plate holder. The background signal was measured in 293T cells not expressing S protein and subtracted to define a specific signal and a cutoff for positivity.

To establish the specificity of the S-Flow assay, we first analyzed a series of 40 human serum samples collected before 2019, from the Institut Pasteur biobank [Investigation Clinique et Accès aux Ressources Biologiques (ICAReB)]. All human serum samples were negative for anti–SARS-CoV-2 S protein antibodies (fig. S2), suggesting that antibodies against other coronaviruses circulating in France were not detected with this assay. We then measured the sensitivity of the assay by assessing the reactivity of serum samples from up to 29 individuals with COVID-19 hospitalized at Hôpital Bichat (table S1). An example of the binding of antibodies from two samples (B1 and B2) is shown in fig. S2B. Serial serum dilutions enabled the determination of antibody titers of 24,600 and 2700 for B1 and B2, respectively (fig. S2B). The median fluorescence intensity (MFI) of the signal decreased with the dilution, showing that MFI, in addition to indicating the percent positive cells, also provided a measurement of the quantity of specific antibodies. We selected a single dilution (1:300) to analyze serum samples from nine individuals with COVID-19 (B1 to B9) (fig. S2C and table S1). We observed an increase in IgG response over time, with seropositivity appearing 6 days after symptom onset. We observed similar patterns with the IgM and IgG antibody responses (fig. S2D). The absence of an earlier IgM response may have been due to the lower sensitivity of the fluorescent anti-IgM antibodies used for detection or to a short delay between the IgM and IgG responses, which has been observed in those with COVID-19. Addressing this question will require the analysis of serum from a greater number of individuals. We also tested a fluorescent anti-whole Ig antibody, but it did not prove more sensitive than the fluorescent anti-IgG antibody. We thus tested serum samples from the different cohorts with the fluorescent anti-IgG antibody.

### Measuring anti–SARS-CoV-2 antibodies in human serum samples with the luciferase immunoprecipitation system assay

The fourth assay that we used, termed LIPS (luciferase immunoprecipitation system), is based on antigens made of viral proteins (or domains) fused to nanoluciferase (fig. S3). The objective was to develop an assay able to test serum samples from diverse cohorts and evaluate the range of antibody responses against a set of viral proteins or domains. The goal was to select the best antigens for high-throughput binding assays. Each antigen was used at the same molar concentration based on standardization of the amount of antigen engaged in each reaction by luciferase activity. This enabled easy direct comparisons of the antibody responses against each antigen. A panel of 10 different S- and N-derived antigens was first evaluated using a set of 34 pre-pandemic human serum samples as well as serum samples from six hospitalized individuals with COVID-19 (fig. S3). Serum samples were obtained from two patients with COVID-19 at three different time points. The strongest signals in the sera from patients with COVID-19 compared to the background signal in pre-pandemic sera were identified as being elicited by S1, S2, and N (C-terminal domain) antigens (fig. S3). Additional investigations on a limited panel of serum samples from mildly symptomatic individuals with COVID-19 showed that antibody responses to S2 were similar to full S protein antibody responses evaluated by the S-Flow assay regarding the diagnostic sensitivity and quantitative antibody response (fig. S3). To avoid redundancy, we focused the LIPS analysis on N antigen because of the sensitivity of this assay for detecting an intracellular viral protein not targeted by neutralizing antibodies, and on S1 antigen, which is thought to be targeted by most neutralizing antibodies. To establish the specificity of the LIPS assay, we first analyzed the same series of 40 serum samples used for the S-Flow assay and found all of the serum samples to be negative for anti–SARS-CoV-2 antibodies (fig. S3). We also measured the kinetics of antibody production in the same longitudinal set of serum samples from five patients with COVID-19 (Fig. 2 and Table 2). We observed an increase in antibody response over time, with seropositivity appearing 7 to 10 days after symptom onset. The protein A/G beads used for precipitation of the immune complexes did not bind efficiently to IgM or IgA antibodies.

**Table 2:** Characteristics of the four cohorts.

**Cohorts**	***n***	**Samples**	**Date**	**Area**	**COVID-19**
Pre-pandemic individuals	491	491	2017–2019	France	Naïve
Hospitalized patients with COVID-19	51	161	January to March 2020	Paris, France	Confirmed
Mildly symptomatic individuals	209	209	3–4 March 2020	Crépy-en-Vallois, France	Suspected
Healthy blood donors	200	200	20–24 March 2020	Lille, France	Unknown

### Characteristics of COVID-19 cohorts

We screened different cohorts of individuals with COVID-19 to evaluate the performance of the four serological assays and their corresponding antigens (Table 2). We first used sera from up to 491 pre-pandemic healthy individuals collected before 2019 to assess the specificity of the assays. We then measured antibody amounts in serum from 51 hospitalized patients with COVID-19 at Hôpital Bichat in Paris to determine the sensitivity of the tests and analyze the kinetics of seroconversion. The clinical and virological characteristics of four of these patients have been described (*24*). We next studied the prevalence of anti–SARS-CoV-2 antibodies in a cohort of mildly symptomatic individuals suspected of having COVID-19 in the city of Crepy-en-Valois in Oise (15,000 inhabitants). Mildly symptomatic individuals were defined as having experienced mild signs compatible with COVID-19 (fever, cough, or dyspnea). On 24 February 2020, a staff member from a high school in Crepy-en-Valois was admitted to a hospital in Paris with confirmed SARS-CoV-2 infection. On 3 to 4 March 2020, students from the high school, parents of the students, teachers, and staff were invited to participate in an epidemiological investigation around this case. A total of 209 blood samples were collected from individuals reporting mild signs, without performing a SARS-CoV-2 reverse transcription quantitative PCR (RT-qPCR) diagnostic test. Last, we tested 200 serum samples from healthy blood donors from the Etablissement Français du Sang (EFS) in Lille. The blood samples were donated in two cities, Clermont (10,000 inhabitants) on March 20 and Noyon (13,000 inhabitants) on March 24, each located about 60 km from Crepy-en-Valois.

### Comparison of the serological assays and estimation of seroprevalence in different subpopulations

**Fig. 1:**
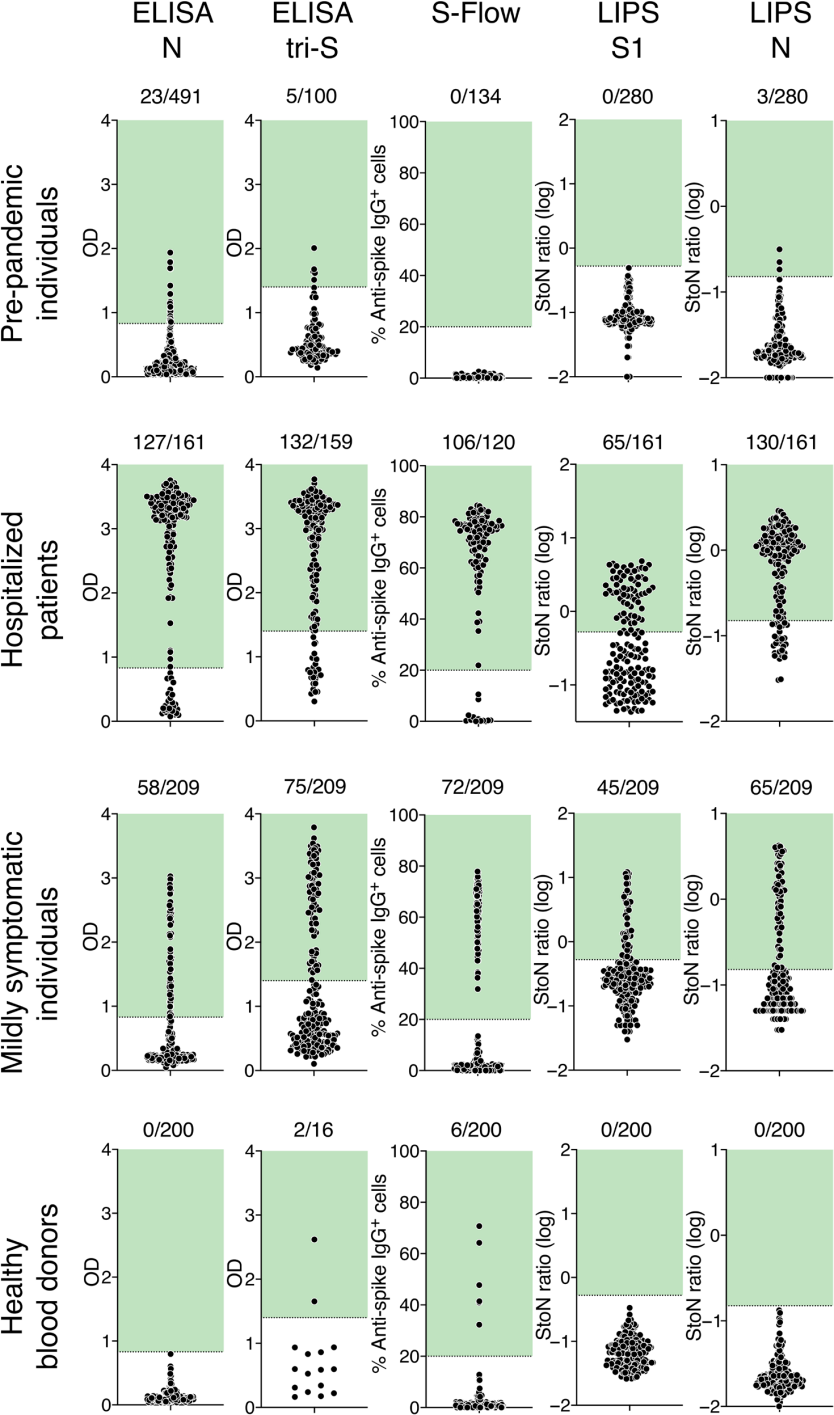
Serological survey of SARS-Cov-2 antibodies in human serum samples. Four serological assays were used to detect anti–SARS-Cov-2 antibodies in serum samples: (**top row**) individuals sampled between 2017 and 2019 (before pandemic), (**second row**) hospitalized cases with confirmed COVID-19, (**third row**) mildly symptomatic individuals from the Crépy-en-Vallois pandemic cluster with suspected COVID-19, and (**bottom row**) healthy blood donors. ELISA N and ELISA tri-S assays are conventional ELISAs using either N protein or the trimeric ectodomain of S protein as antigens. S-Flow is an assay detecting antibodies bound to cells expressing S protein by flow cytometry. The LIPS S1 and N assays detect either S1 or N protein fused to luciferase by immunoprecipitation. Pre-pandemic serum samples were used to determine the cutoff for each assay, which is indicated by a dotted line and a green area. The two ELISA assays were set to 95% specificity; the specificity of the S-Flow assay and LIPS assay was 99%. The number of positive samples is indicated. Each dot represents one sample. OD, optical density; StoN, signal-to-noise ratio.

Figure 1 shows results obtained with sera from each category of individuals (before pandemic, hospitalized patients with COVID-19, mildly symptomatic individuals, and healthy blood donors). The pre-pandemic serum samples served as negative controls. With the four serological assays, signals were consistently negative (S-Flow assay and LIPS S1 assay) or low (the two ELISAs and the LIPS N assay) with the pre-pandemic serum samples (Fig. 1). This suggested that previous exposure to human seasonal coronaviruses associated with the common cold [such as human coronavirus (HCoV)–OC43, HCoV-229E, HCoV-HKU-1, or HCoV-NL63] did not induce an obvious cross-reaction with our assays. This was expected because these prevalent coronaviruses are only distantly related to SARS-CoV-2 at the protein level. For each assay, we established cutoff thresholds. For the ELISA N assay, the cutoff was set at 95% percentile for 491 pre-pandemic serum samples corresponding to 95% specificity. For the ELISA tri-S assay, the cutoff was established on the basis of ROC analyses (fig. S1) and corresponded to the mean ± 2.2 SDs of the 100 pre-pandemic serum samples analyzed (95% specificity). For the S-Flow assay, we established a cutoff that corresponded to a signal >20% of cells positive by flow cytometry. For the LIPS N assay, the cutoff was based on internal controls (99% specificity). The S-Flow and LIPS S1 assay cutoffs eliminated all pre-pandemic serum samples analyzed (100% specificity).

**Fig. 2:**
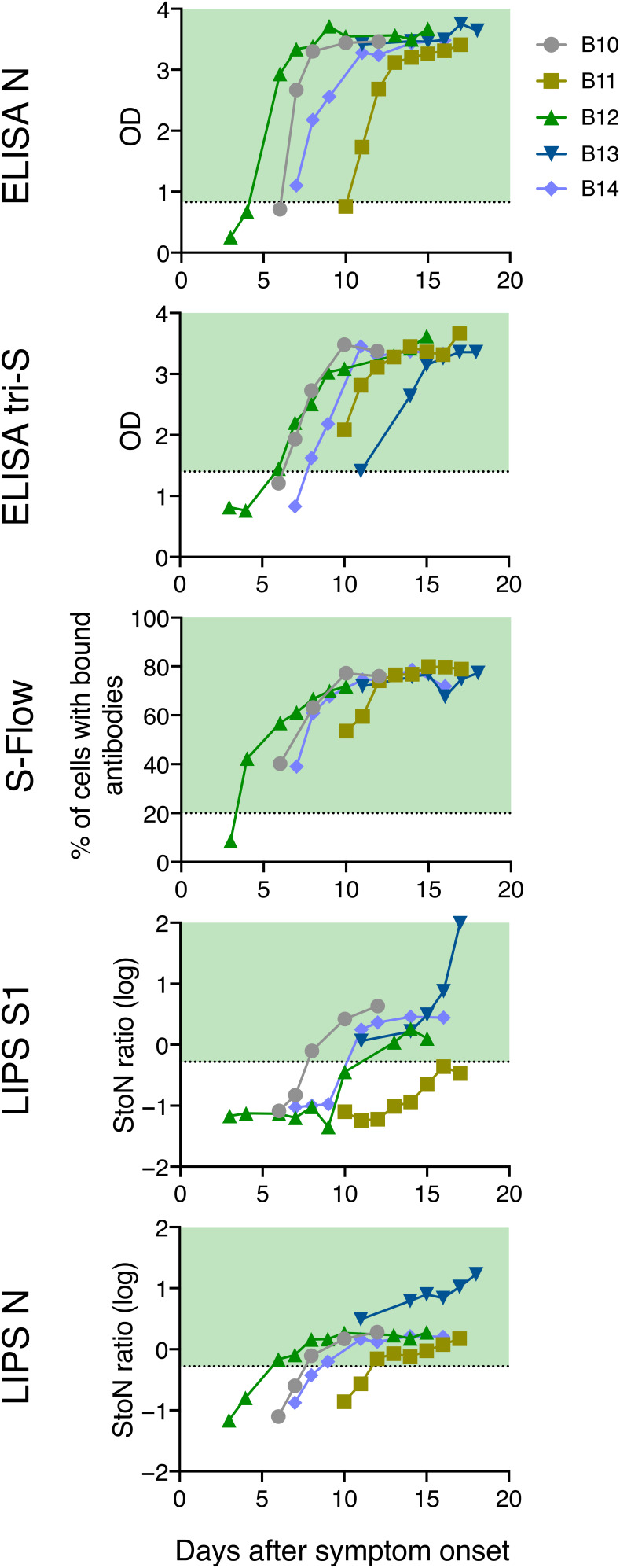
Antibody detection in serum samples from five hospitalized patients with COVID-19. The kinetics of seroconversion in serum samples from five hospitalized patients with COVID-19 (B10 to B14) were measured by four different serological assays. At least five longitudinal serum samples were collected for each patient up to 20 days after symptom onset. All patients were admitted to the intensive care unit. Each line represents one patient. Dotted lines and green areas indicate cutoff for positivity in the seroprevalence assays.

Having established these cutoff values, we analyzed serum samples from 51 patients with COVID-19 at Hôpital Bichat. Some of these patients were analyzed at different time points, representing a total of up to 161 serum samples. The percentage of positive samples varied between 65 and 72%, with a mean of 69%. The fact that not all patients were seropositive reflected the various sampling times from each individual. To study more precisely the kinetics of seroconversion and the dynamics of the humoral response, we selected five hospitalized patients with COVID-19 with more than five longitudinal serum samples and known dates of symptom onset (Fig. 2). In these patient serum samples, seroconversion was detected between 5 and 10 days after symptom onset with ELISA N, LIPS N, ELISA tri-S, and S-Flow assays. Antibody binding intensities increased over time and rapidly reached a plateau (Fig. 2). The LIPS S1 assay became positive with slower kinetics, and one of the patient serum samples remained just below the cutoff. For some patient serum samples, the LIPS N and ELISA N signals appeared before the LIPS S1 and ELISA tri-S signals, which suggested different kinetics of N responses and S/S1 responses independently of the sensitivity of the test.

We then tested the 209 serum samples obtained in Oise from suspected cases of COVID-19 with mild clinical signs. Positivity rates varied from 27 to 36% among the assays, with a mean of 32% (Fig. 1 and Table 3). This range of variation was more marked than with hospitalized COVID-19 patient samples, most likely because mildly symptomatic individuals had lower viral loads than did those requiring hospitalization and consequently might have generated lower quantities and different patterns of antibodies. The fact that only one-third of the mildly symptomatic individuals tested positive suggested that some of them may not have seroconverted by the time the sample was taken or other viruses or environmental causes were responsible for the reported symptoms. These results are in line with a recent study performed on a similar cohort of 1340 individuals with mild symptoms (*25*). This study showed that only 40% of suspected mild cases had antibodies, whereas almost 100% of RT-qPCR–confirmed mild cases of COVID-19 were seropositive (*25*).

**Table 3:** SARS-CoV-2 seroprevalence in hospitalized patients with COVID-19, mildly symptomatic individuals, and healthy blood donors.

**Cohort**	**ELISA N**	**ELISA tri-S**	**S-Flow**	**LIPS S1 + N**	**Antibody prevalence**
Pre-pandemic individuals (specificity)	23/491 (95%)	5/100 (95%)	0/134 (100%)	3/280 (99%)	
Hospitalized patients (seroprevalence)	33/51 (65%)	35/51 (69%)	21/29 (72%)	35/51 (69%)	69% (65–72%)
Mildly symptomatic individuals	56/209 (27%)	75/209 (36%)	73/209 (35%)	68/209 (32%)	32% (27–36%)
Healthy blood donors	0/200	2/16	6/200	0/200	3%

We next examined SARS-CoV-2 seroprevalence in serum samples collected from healthy blood donors in Oise on 20 to 24 March 2020. Eligibility criteria for blood donation included an absence of recent signs of infection or antibiotic treatment. These healthy blood donors were seronegative by the ELISA N and LIPS assays. With the S-Flow assay, six blood donors were positive, including two with a strong signal. These 6 seropositive blood donors and another 10 seronegative blood donors were then tested with the ELISA tri-S assay, and only the 2 strong responders scored positive. Therefore, the positivity rate in this cohort was low (1 to 3% using the two most sensitive serological assays). This suggested that at the time of blood collection, SARS-CoV-2 had not circulated to a large extent around the initial cases in the city of Crepy-en-Valois. It is also likely that asymptomatic SARS-CoV-2 infection induced low or delayed seroconversion.

### Correlations among the four serological assays

**Fig. 3:**
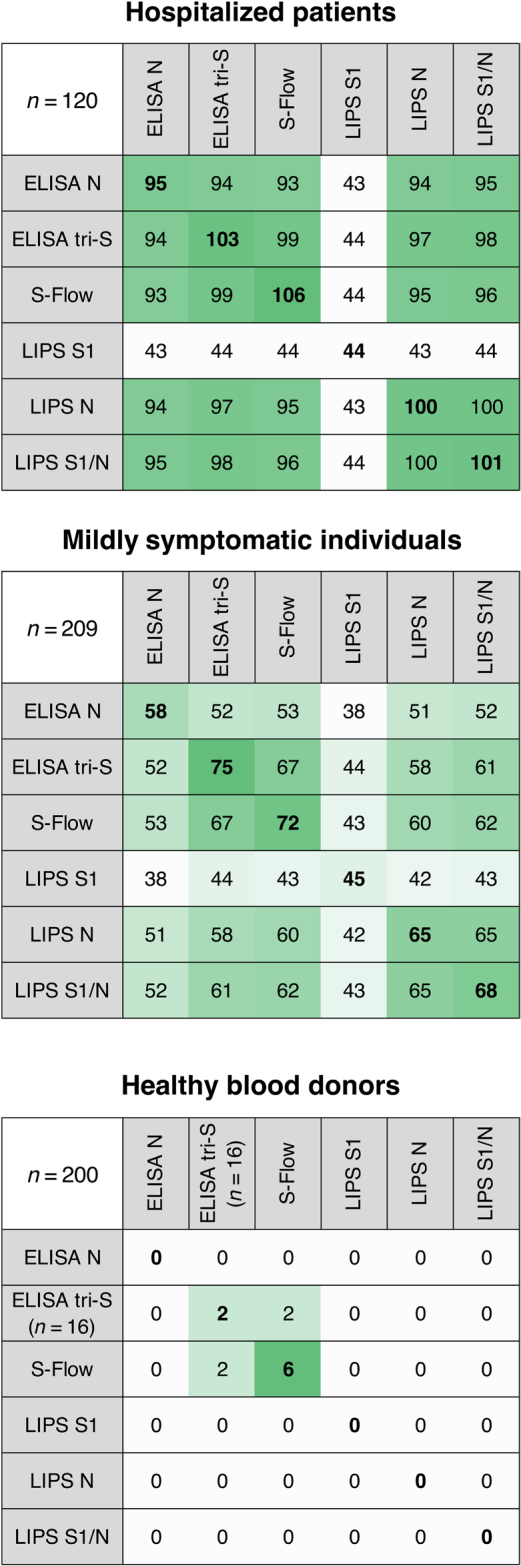
Comparison of positive serum samples. The number of positive serum samples detected by each serological assay is shown for the three cohorts: hospitalized patients with COVID-19, mildly symptomatic individuals, and healthy blood donors. Correspondence of the positive results is shown among the four assays. For a given assay, each row indicates the number of positive samples that were also positive with the other three assays. Bold numbers indicate the number of positive samples for a given assay. The number of positive samples is color-coded: White corresponds to lower numbers, and green corresponds to higher numbers.

We performed a side-by-side comparison of the four serological assays using our three cohorts: pre-pandemic individuals, hospitalized patients with COVID-19, and mildly symptomatic individuals. For a given assay, we first scored the number of positive serum samples measured with the other assays (Fig. 3). With hospitalized patient sera, similar numbers of positive samples were obtained with the four assays, with the exception of the LIPS S1 assay, confirming that this assay was less sensitive probably because it does not detect antibodies targeting other S protein domains (Fig. 3). However, combining the LIPS S1 assay and N assay results gave similar detection rates compared to the other three assays. With the cohort of mildly symptomatic individuals, the S-Flow and ELISA tri-S assays yielded similar results and higher detection rates than the other two tests (Fig. 3). Among the healthy blood donors, positive cases were only detected with these two assays.

**Fig. 4:**
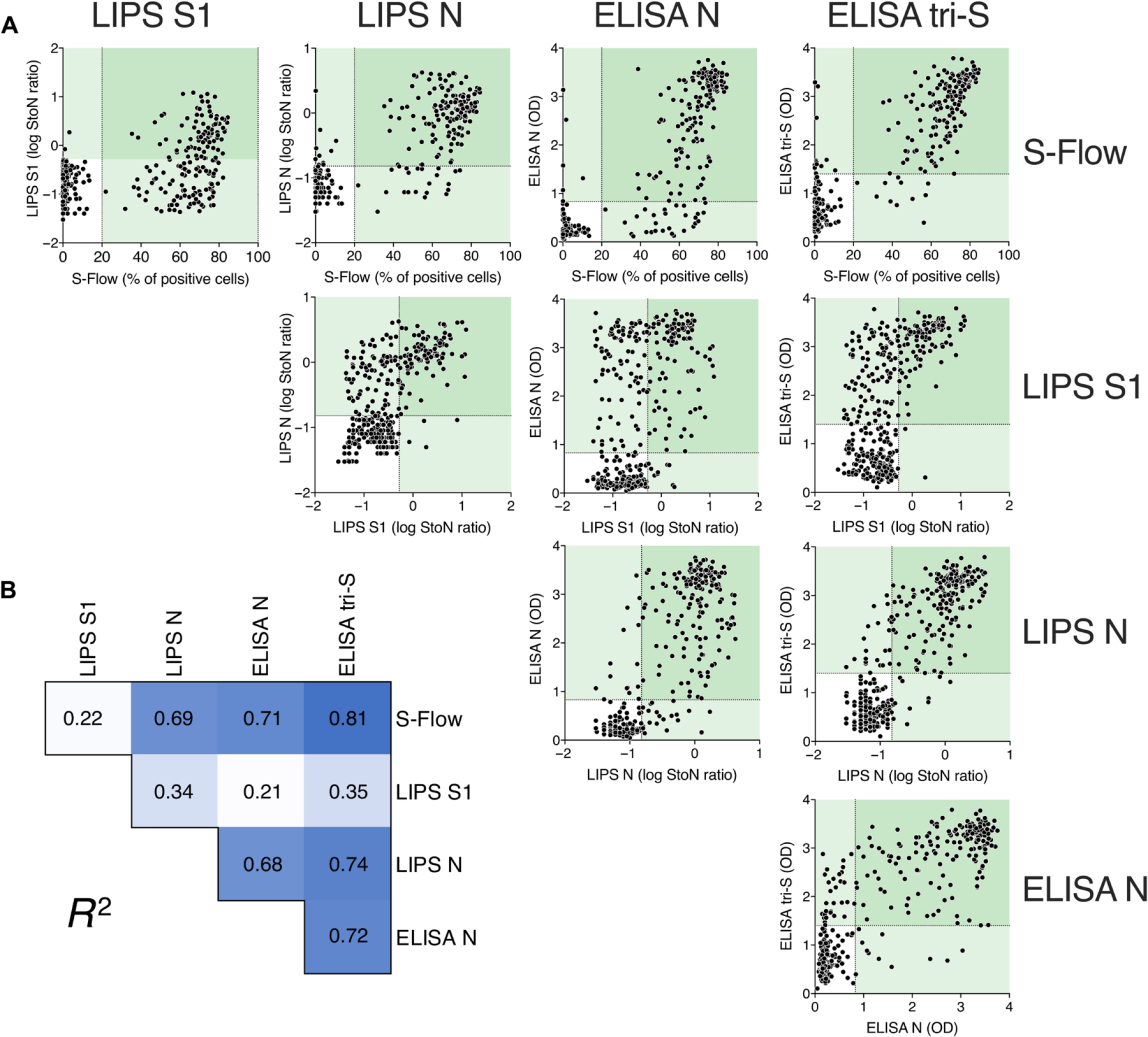
Correlations among the four serological assays. To compare the four serological assays, results from serum samples from mildly symptomatic individuals and hospitalized patients with COVID-19 (*n* = 329) were pooled. (**A**) Results obtained with one assay were correlated with those of the other three assays. Dotted lines indicate assay cutoff values for positivity. Values in pale green areas are positive in one assay, and values in darker green areas are positive in two assays. Each dot represents one study participant. (**B**) Pearson correlation coefficient (*R*^2^) of each comparison is shown. *R*^2^ values are color-coded, with white corresponding to the lowest value and dark blue corresponding to the highest value. All correlations are significant (*P* < 0.0001).

We then pooled results obtained with samples from all three cohorts and calculated correlation rates for each serological assay (Fig. 4). The dot plots indicate that serum samples with high antibody concentrations were detected by all four assays. Important differences were observed among the assays with serum samples with low antibody concentrations, reflecting both the choice of the antigens and the different sensitivities of the assays.

### SARS-CoV-2 microneutralization and pseudovirus neutralization assays

We then evaluated the presence of neutralizing antibodies in the sera of SARS-CoV-2–infected individuals. Various tests have already been established for measuring antibody neutralizing capacity (*6*, *8*, *19*, *21*), so we focused on two tests. The first was a microneutralization assay using SARS-CoV-2 virions. This reference method was based on virus incubation with serial dilutions of the serum sample to be tested and evaluation of virus titers in Vero-E6 cells. We developed a second lentivirus-based pseudotype neutralization assay (fig. S4A). Lentivirus particles coated with SARS-CoV-2 S protein and encoding a green fluorescent protein (GFP) reporter gene were pretreated with dilutions of the serum sample to be tested. The lentivirus particles were then incubated with target cells (293T cells transiently expressing ACE2 and TMPRSS2 protease), and the fluorescent signal was measured after 48 hours. A pilot experiment with 14 serum samples from hospitalized patients with COVID-19 demonstrated strong neutralizing activity with 8 of the 14 serum samples (fig. S4, B and C). As a control, we used lentivirus particles coated with an irrelevant viral protein (VSV-G), which were insensitive to the same 14 serum samples (fig. S4C). We also tested as a proof of concept the neutralization activity of the first 12 serum samples of the cohort of mildly symptomatic individuals with suspected COVID-19 (fig. S4D). A strong correlation was observed between the results of the microneutralization assay with SARS-CoV-2 virions and the pseudovirus neutralization assay (fig. S4E).

**Fig. 5:**
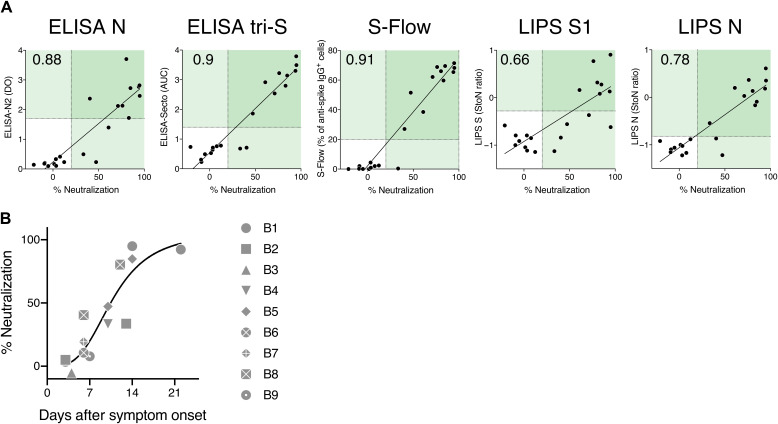
Virus neutralizing activity in human serum samples. (**A**) Virus-neutralizing activity (dilution 1:100) of 12 serum samples from the mildly symptomatic cohort of individuals with suspected COVID-19 (C1 to C12) and 9 serum samples from hospitalized patients with COVID-19 (B1 to B9). Virus-neutralizing activity was determined by the pseudovirus neutralization assay and compared to serology data obtained with the four serological assays. Numbers in the top left quadrant indicate the Spearman correlation coefficient, *r*. All correlations are significant (*P* < 0.0001). (**B**) Neutralization activity of serum samples (B1 to B9) from hospitalized patients with COVID-19 was plotted against days after symptom onset. The black line corresponds to a nonlinear fit of the data.

The reference microneutralization assay was labor intensive and required access to a Biosafety Level 3 (BSL3) facility. We thus performed a pilot correlative analysis between the four serological assays and the pseudovirus neutralization assay (Fig. 5A). This analysis was performed with serum samples from 9 hospitalized patients with COVID-19 and 12 mildly symptomatic individuals. A strong correlation was observed with the ELISA N, ELISA tri-S, S-Flow, and LIPS N assays, with a similar but less marked trend with the LIPS S1 assay. We also determined by linear regression the association between the intensity of antibody binding and pseudovirus neutralization. A neutralization activity >80% was associated with the following signals: ELISA N (>2.37 OD), ELISA tri-S (>2.9 OD), S-Flow (>60% positive cells), and LIPS N (>0.049 signal-to-noise ratio). With this level of neutralization activity, the LIPS S1 assay mainly gave positive responses and a few responses below the cutoff. In nine hospitalized patients with COVID-19, the neutralization activity increased over time: It was first detectable at day 5 after symptom onset and reached 50% 7 to 14 days after symptom onset and 80 to 100% 14 to 21 days after symptom onset (Fig. 5B). These pilot experiments were performed with a limited number of serum samples originating from individuals with mild, severe, or critical symptoms. It will be important to increase the number of mildly symptomatic individuals tested and to evaluate whether asymptomatic seropositive individuals have antibodies that exhibit virus neutralization activity.

## DISCUSSION

We have used four different serological assays to detect anti–SARS-CoV-2 antibodies in human serum samples from several different cohorts. The first two assays were ELISAs detecting anti-N and anti-S protein antibody responses. The S-Flow assay allowed us to identify and measure antibodies binding to all domains and conformations of the SARS-CoV-2 S protein expressed at the cell surface. The LIPS assays targeted different domains of S and N and enabled the detailed profiling of the humoral responses. We have evaluated the performance of the four serological assays and compared their results with two virus neutralization assays, a reference microneutralization assay and a pseudovirus-based neutralization assay.

Each serological assay showed advantages and drawbacks. ELISAs are widely used in commercial tests, can be easily performed in routine diagnostic laboratories, and can test large quantities of samples. In the ELISAs that we performed, the high ODs that we observed with a limited number of negative control samples may have been due to the presence of antibodies directed against antigens from other sources, including from other coronaviruses, that displayed cross-reactivity with SARS-CoV-2 proteins. These outlier negative control samples were not found to be positive with the other assays. The S-Flow assay captured all anti–SARS-CoV-2 S protein antibodies and provided excellent sensitivity but required access to a cell culture system and flow cytometry equipment. Thus, it is less well adapted to high-throughput screening of large numbers of samples. The LIPS assay enabled the testing of different target antigens in a liquid-phase assay, preserving as much as possible the conformational epitopes of the antigens. It appeared to be as sensitive as ELISA and the S-Flow assay for some of the antigens tested but did require access to a bioluminescence detection instrument. The two virus neutralization assays required cell culture facilities, with the microneutralization assay using infectious SARS-CoV-2 necessitating access to a BSL3 facility. In contrast, the pseudovirus neutralization assay could be adapted for high-throughput screening without the need for a BSL3 facility.

Serological tests are complementary to viral detection by RT-qPCR for diagnostic purposes for those being tested for COVID-19. Results from our study and others indicate that in severe cases of COVID-19 when patients are hospitalized, seroconversion is detectable as soon as 5 to 14 days after symptom onset and that antibody concentrations increase rapidly reaching a plateau (*6*, *7*, *13*–*15*). In such cases, antibody can reach high titers, and different assays give similar results as we show here. Detection of anti-N and anti-S antibody responses demonstrated similar rates of seroconversion, whereas the anti-S1 antibody response was delayed. The anti-N antibody response appeared slightly more rapidly than did antibody responses to S/S1 for a given type of test. If confirmed, this could be of interest for developing routine diagnostic tests.

At the population level, serological tests are being used in serological surveys to identify persons who have been infected with SARS-CoV-2. Regarding the identification of mildly symptomatic or asymptomatic individuals, we consistently observed a roughly similar seroprevalence, with different sensitivities depending on the assay. ELISA tri-S, S-Flow, and the combined LIPS S1 + N assays gave slightly higher detection rates than did the ELISA N assay. Combining the ELISA N and S assays may also increase the sensitivity of antibody detection.

One limitation of our study is that the group of 209 mildly symptomatic individuals sampled as part of an outbreak investigation included both individuals who had been infected with SARS-CoV-2 and individuals who were infected by other respiratory viruses. We do not know the virological status of these 209 individuals and therefore cannot evaluate the sensitivity of our different assays using samples from these suspected cases. Our study was not intended to compare RT-qPCR and antibody testing, but rather to evaluate the seroprevalence of SARS-CoV-2 in a region of France where cluster cases were identified. The short time frame of the sampling also allowed us to compare the performance of the four different serological assays at a given date and within a limited geographical region. We recently reported that among a cohort of 160 hospital staff in Strasbourg (France) with mild symptoms who were all positive by RT-qPCR for SARS-CoV-2 infection, a rapid immunodiagnostic commercial test detected antibodies in 153 (95.6%) of these samples and the S-Flow assay detected antibodies in 159 (99.4%) of these samples (*26*). The sensitivity of the S-Flow assay is thus >99%. Antibody titers increased over time for at least up to 28 to 41 days after symptom onset (*26*). It will be useful to perform a similar analysis with the other three assays to further assess their performance with serum samples from individuals diagnosed with SARS-CoV-2 infection.

Another limitation of our study is that we tested the serum samples at only one serum dilution, with the chosen dilution varying based on the test. This dilution was selected to obtain optimal sensitivity and specificity. Future work with serial dilutions of serum samples will allow a precise quantification of antibody titers.

What is the extent of the neutralizing immune response in infected individuals with different disease severity? In 175 convalescent patients with mild symptoms of COVID-19, neutralizing antibodies were detected from 10 to 15 days after symptom onset in a large fraction of patients (*20*). The titers of neutralizing antibodies correlated with the titers of anti–SARS-CoV-2 S protein antibodies (targeting S, RBD, and S2 regions) (*20*). In our previous study of 160 hospital staff with mild symptoms of COVID-19, we also observed a neutralizing activity in serum samples from the large majority of cases (*26*). A critical question concerns the detection of antibodies and their neutralization potential in asymptomatic individuals with COVID-19 and, more generally, what the correlates of protection are. Recent reports indicate that asymptomatic individuals with COVID-19 mount a neutralizing humoral response that is lower than that observed in symptomatic persons or hospitalized patients with COVID-19 (*27*) (*28*) (*29*). In our pilot study of serum samples from 200 healthy blood donors, the ELISA N and LIPS S1 + N assays were negative for anti–SARS-CoV-2 antibodies, whereas six serum samples scored positive with the S-Flow assay. When samples were reanalyzed with the ELISA tri-S assay, two of the six samples were positive. These results indicate that the most sensitive assays are required for identification of asymptomatic SARS-CoV-2–infected individuals, but this should not be at the expense of specificity because this could considerably affect the predictive value of positive results in low-prevalence areas.

Neutralizing antibodies play a major role in preventing reinfection by many viruses. A key issue is the relationship between in vivo protection and the extent that antibody binds to and neutralizes the virus. We compared our serological assays to the virus microneutralization and pseudovirus neutralization assays in a limited number of samples from hospitalized patients with COVID-19 and mildly symptomatic individuals. We observed a strong correlation between the extent of the anti–SARS-CoV-2 full-length S protein antibody response (and even the anti-N protein antibody response) and the virus neutralization capacity of the serum samples. We are currently examining whether antibody titers and which viral proteins best correlate with virus neutralization capacity in samples from mildly symptomatic or asymptomatic seropositive individuals. Answering this question will help in determining whether a serological high-throughput assay may serve as a surrogate to estimate protection at the individual or population level. This is an important parameter to understand and will be key for modeling the dynamics and evolution of the pandemic and for defining serological tools for controlling the spread of infection at the population level.

Non-neutralizing antibodies, or neutralizing antibodies at suboptimal doses, can contribute to antibody-dependent enhancement of infection. Antibody-dependent enhancement exacerbates disease caused by the related coronaviruses feline coronavirus, MERS-CoV, and SARS-CoV (*30*–*33*). Antibody-dependent enhancement might also play a deleterious role in COVID-19. The various techniques described here will be important for determining the serological status of individuals or populations and for establishing potential immune correlates of COVID-19 facilitation or protection.

## MATERIALS AND METHODS

### Study design

The objective of this study was to develop serological assays to assess the presence of anti–SARS-CoV-2 antibodies in serum samples from different groups of individuals. Four assays measuring antibody concentrations and two assays measuring their neutralization activity against SARS-CoV-2 were implemented. The performance of the assays was evaluated on sera obtained from individuals before pandemic, hospitalized patients with COVID-19, mildly symptomatic individuals with suspected COVID-19, and healthy blood donors. Sample sizes were chosen empirically to ensure adequate statistical power. Investigators were not blinded with respect to the origin of the samples. For the validation of the tests, each serum sample was measured multiple times as detailed in the figure legends. For analysis of the cohorts, each serum sample was analyzed two or three times. Serum samples with discordant results between tests were reanalyzed to confirm their status. All valid measurements were included in our analysis. No outliers were excluded. Primary data are provided in the figures or the Supplementary Materials.

### Characteristics of cohorts

Pre-pandemic sera originated from two healthy donor sources: 200 serum samples from the Diagmicoll cohort collection of ICAReB (*34*) approved by Comité de Protection des Personnes Ile-de-France and sampled before November 2019, and 200 anonymized serum samples from healthy blood donors recruited in March 2017 at the Val d’Oise sites of EFS (the French blood agency). The ICAReB platform (BRIF code n°BB-0033-00062) of Institut Pasteur collects and manages bioresources following ISO (International Organization for Standardization) 9001 and NF S 96-900 quality standards (*34*).

Serum samples from COVID-19 cases were obtained from Hôpital Bichat–Claude-Bernard as part of the French COVID-19 cohort. Some of the patients were previously described (*24*). Each participant provided written consent to participate in the study, which was approved by the regional investigational review board (Comité de Protection des Personnes Ile-de-France VII, Paris, France) (ID RCB: 2020-A00256-33) and performed according to European guidelines and the Declaration of Helsinki.

Serum samples were obtained from mildly symptomatic individuals in the following way. On 24 February 2020, a patient from Crepy-en-Valois (Oise region, northern France) was admitted to a hospital in Paris with confirmed SARS-CoV-2 infection. As part of an epidemiological investigation around this case, a cluster of COVID-19 cases was identified at a high school with an enrolment of 1200 pupils. On 3 to 4 March 2020, students at the high school, their parents, teachers, and staff (administrative staff, cleaners, and catering staff) were invited to participate in the investigation. Study participants (with the help of their parents in the case of students) completed a questionnaire that covered sociodemographic information, underlying medical conditions, history of respiratory symptoms back to 13 January 2020, and a history of COVID-19 diagnosis before this investigation. A 5-ml blood sample was taken from all study participants who had experienced respiratory symptoms since 13 January 2020. A total of 209 individuals were recruited to the study, and 203 completed the questionnaire through a live interview. The characteristics of the study participants are presented in table S2. One self-registered symptom (described in table S2) was enough for inclusion in the cohort. This study was registered with ClinicalTrials.gov (NCT04325646) and received ethical approval by the Comité de Protection des Personnes Ile-de-France III. Informed consent was obtained from all study participants.

Samples from healthy blood donors were collected in accordance with local ethical guidelines by EFS (Lille, France) in Clermont (Oise) on 20 March 2020 and Noyon (Oise) on 24 March 2020, two cities located 60 km from Crepy-en-Valois. All sera were heat-inactivated for 30 to 60 min at 56°C, aliquoted, and conserved at 4°C for short-term use or frozen.

### Characteristics of the four serological assays

#### ELISA N assay

A codon-optimized nucleotide fragment encoding full-length nucleoprotein was synthesized and cloned into pETM11 expression vector (European Molecular Biology Laboratory). The His-tagged SARS-CoV-2 N protein was bacterially expressed in *E. coli* BL21 (DE3) and purified as a soluble dimeric protein by affinity purification using a Ni-NTA Protino column (Macherey Nagel) and gel filtration using a HiLoad 16/60 Superdex 200 pg column (GE Healthcare). Ninety-six–well ELISA plates were coated overnight with N in phosphate-buffered saline (PBS) (50 ng per well in 50 μl). After washing four times with PBS–0.1% Tween 20 (PBST), 100 μl of diluted sera (1:200) in PBST–3% milk was added and incubated for 1 hour at 37°C. After washing three times with PBST, plates were incubated with 8000-fold diluted peroxidase-conjugated goat anti-human IgG (Southern Biotech) for 1 hour. Plates were revealed by adding 100 μl of horseradish peroxidase (HRP) chromogenic substrate [3,3′,5,5′-tetramethylbenzidine (TMB), Eurobio Scientific] after three washing steps in PBST. After 30-min incubation, ODs were measured at 405 nm (OD_405nm_). OD measured at 620 nm was subtracted from values at 405 nm for each sample.

#### ELISA tri-S assay

A codon-optimized nucleotide fragment encoding a stabilized version of the SARS-CoV-2 S ectodomain (amino acids 1 to 1208) followed by a foldon trimerization motif and tags (8×HisTag, StrepTag, and AviTag) was synthesized and cloned into pcDNA3.1/Zeo(+) expression vector (Thermo Fisher Scientific). Trimeric S (tri-S) glycoproteins were produced by transient cotransfection of exponentially growing Freestyle 293-F suspension cells (Thermo Fisher Scientific, Waltham, MA) using polyethylenimine (PEI) precipitation method as previously described (*35*). Recombinant tri-S proteins were purified by affinity chromatography using the Ni Sepharose excel resin according to the manufacturer’s instructions (Thermo Fisher Scientific). Protein purity was evaluated by in-gel protein silver staining using Pierce Silver Stain kit (Thermo Fisher Scientific) after SDS–polyacrylamide gel electrophoresis in reducing and nonreducing conditions using NuPAGE 3 to 8% tris-acetate gels (Life Technologies). High-binding 96-well ELISA plates (Costar, Corning) were coated overnight with 125 ng per well of purified tri-S proteins in PBS. After washings with PBST, plate wells were blocked with PBS–1% Tween 20–5% sucrose–3% milk powder for 2 hours. After PBST washings, 1:100-diluted sera in PBST–1% bovine serum albumin (BSA) and seven consecutive 1:4 dilutions were added and incubated for 2 hours. After PBST washings, plates were incubated with 1000-fold diluted peroxidase-conjugated goat anti-human IgG/IgM/IgA (Jackson ImmunoResearch, 0.8 μg/ml final) for 1 hour. Plates were revealed by adding 100 μl of HRP chromogenic substrate (ABTS solution, Euromedex) after PBST washings. ODs were measured at 405 nm (OD_405nm_) after a 30-min incubation. Experiments were performed in duplicate at room temperature and using a HydroSpeed microplate washer and a Sunrise microplate absorbance reader (Tecan, Männedorf, Switzerland). AUC values were determined by plotting the log_10_ of the dilution factor values (*x* axis) required to obtain OD_405nm_ values (*y* axis). AUC calculation and ROC analyses were performed using GraphPad Prism software (v8.4.1, GraphPad Prism Inc.).

#### S-Flow assay

Human embryonic kidney (HEK) 293T (referred as 293T) cells were from the American Type Culture Collection (ATCC) (ATCC CRL-3216) and tested negative for mycoplasma. Cells were split every 2 to 3 days using Dulbecco’s modified Eagle’s medium (DMEM) supplemented with 10% fetal calf serum and 1% penicillin-streptomycin (complete medium). A codon-optimized version of the SARS-Cov-2 S gene (GenBank: QHD43416.1) (*1*) was transferred into the phCMV backbone (GenBank: AJ318514) by replacing the VSV-G gene. 293T cells were transfected with S or a control plasmid using Lipofectamine 2000 (Life Technologies). One day after, transfected cells were detached using PBS-EDTA and transferred into U-bottom 96-well plates (50,000 cells per well). Cells were incubated at 4°C for 30 min with sera (1:300 dilution, unless otherwise specified) in PBS containing 0.5% BSA and 2 mM EDTA, washed with PBS, and stained using either anti-IgG Alexa Fluor 647 (Thermo Fisher Scientific) or anti-IgM [phycoerythrin (PE) by Jackson ImmunoResearch or Alexa Fluor 488 by Thermo Fisher Scientific]. Cells were washed with PBS and fixed for 10 min using 4% paraformaldehyde (PFA). Data were acquired on an Attune NxT instrument (Life Technologies). In less than 0.5% of the samples tested, we detected a signal in control 293T cells, likely corresponding to antibodies binding to other human surface antigens. Specific binding was calculated with the following formula: 100 × (% binding on 293T-S − binding on control cells)/(100 − binding on control cells). We generated stably expressing 293T-S cells during completion of this study, which yielded similar results.

#### LIPS assay

Ten recombinant antigens were designed on the basis of the viral genome sequence of the SARS-CoV-2 strain France/IDF0372/2020 (accession no. EPI_ISL_406596) obtained from GISAID database (*36*). Five targeted different domains of S were as follows: Full S1 subunit (residues 1 to 698), N-terminal domain of S1 (S1-NTD, residues 1 to 305), domain connecting the S1-NTD to the RBD (S1-CD, residues 307 to 330 and 529 to 700 connected by a GGGSGG linker), Full S2 subunit (residues 686 to 1208), and S441-685. For constructs that did not contain an endogenous signal peptide (residues 1 to 14), i.e., S1-CD and S2 constructs, an exogenous signal peptide coming from a human kappa light chain (METDTLLLWVLLLWVPGSTG) was added to ensure efficient protein secretion into the media. Five additional recombinant antigens, targeting overlapping domains of N, were designed: Full N (residues 1 to 419), N-terminal domain (residues 1 to 209), C-terminal domain (residues 233 to 419), N120-419, and N111-419. The LIPS assay was designed as described (*37*) with minor modifications. Expression vectors were synthesized by GenScript Company, using as backbone the pcDNA3.1(+) plasmid, with codon usage optimized for human cells. HEK293F cells were grown in suspension and transfected with PEI (PEI-25 kDa, Polysciences Inc., USA). Valproic acid (2.2 mM) was added at day 1 to boost expression. Recombinant proteins were harvested at day 3 in supernatants or crude cell lysates. Luciferase activity was quantified with a Centro XS^3^ LB 960 luminometer (Berthold Technologies, France). A total of 10^8^ luciferase unit (LU) of antigens were engaged per reaction. S1 and C-terminal domain (residues 233 to 419) were selected for analyzing the cohorts. To increase sensitivity, the cohorts were tested at a final dilution of 1:10 of sera.

### Characteristics of the neutralization assays

#### Microneutralization assay

Vero-E6 cells were seeded in a 96-well plate at 2 × 10^4^ cells per well. The following day, 100 TCID_50_ (Fifty-percent tissue culture infectious culture dose) of virus (strain BetaCoV/France/IDF0372/2020) were incubated with serial twofold dilutions of sera, starting from 1:10, in 100 μl of DMEM with trypsin-TPCK [6-(1-tosylamido-2-phenyl) ethyl chloromethyl ketone] at 1 μg/ml to enhance viral infectivity, for 1 hour at 37°C. Mixes were then added to cells and incubated for 2 hours at 37°C. Virus/serum mixes were removed, 100 μl of DMEM + trypsin-TPCK (1 μg/ml) was added, and cells were incubated for 72 hours at 37°C. Virus inoculum was back-titrated in each experiment. Cytopathic effect (CPE) reading was performed by direct observation under the microscope after cell coloration with crystal violet. Microneutralization titers are expressed as the serum dilution for which 50% neutralization is observed.

#### S-pseudotype neutralization assay

Pseudotyped viruses were produced by transfection of 293T cells as previously described (*38*). Briefly, cells were cotransfected with plasmids encoding for lentiviral proteins, a GFP reporter (or a luciferase reporter when specified) and the SARS-CoV-2 S plasmid, or the VSV-G plasmid as a control. Pseudotyped virions were harvested at days 2 and 3 after transfection. Production efficacy was assessed by measuring infectivity or p24 concentration.

293T cells were transiently transfected with ACE2 and TMPRSS2 expression plasmids using Lipofectamine 2000 (Life Technologies) as described above. Twenty-four hours after transfection, cells were detached with PBS-EDTA and seeded in flat-bottom 96-well plates. S-pseudotypes were incubated with sera to be tested (at 1:100 dilution, unless otherwise specified) in culture medium, incubated for 10 min at room temperature, and added on cells. After 48 hours, cells were detached using PBS-EDTA, fixed with 4% PFA, and analyzed on an Attune NxT flow cytometer. The frequency of GFP^+^ cells in each condition was determined using FlowJo v10 software, and neutralization was calculated using the following formula: 100 × (mean of replicates − mean of negative controls)/(mean positive controls − mean of negative controls). S-pseudotypes incubated without serum and medium alone were used as positive and negative controls, respectively. 293T cells stably expressing ACE2 were also used in this assay and yielded similar results. For luciferase-expressing pseudotypes, samples were analyzed with an EnSpire instrument (PerkinElmer).

### Statistical analysis

Flow cytometry data were analyzed with FlowJo v10 software (TriStar). Calculations were performed using Excel 365 (Microsoft). Figures were drawn in Prism 8 (GraphPad Software). Statistical analysis was conducted using GraphPad Prism 8. Statistical significance between different groups was calculated using a two-tailed Mann-Whitney test. Correlations were assessed using Pearson correlation coefficient. *P* < 0.05 was considered statistically significant.

## SUPPLEMENTARY MATERIALS

stm.sciencemag.org/cgi/content/full/scitranslmed.abc3103/DC1 Fig. S1. Seroreactivity of the SARS-CoV-2 tri-S ELISA assay. Fig. S2. Seroreactivity of the S-Flow assay. Fig. S3. Seroreactivity of the SARS-CoV-2 LIPS assays. Fig. S4. Characteristics of the pseudovirus neutralization assay. Table S1. Clinical status of hospitalized patients with COVID-19. Table S2. Characteristics of mildly symptomatic individuals with suspected SARS-CoV-2 infection. Data file S1. Individual-level data for figures.

## Appendix

View/request a protocol for this paper from *Bio-protocol*.
